# Nonphosphorylated tau slows down Aβ_1–42_ aggregation, binds to Aβ_1–42_ oligomers, and reduces Aβ_1–42_ toxicity

**DOI:** 10.1016/j.jbc.2021.100664

**Published:** 2021-04-16

**Authors:** Marten Beeg, Elisabetta Battocchio, Ada De Luigi, Laura Colombo, Carmina Natale, Alfredo Cagnotto, Alessandro Corbelli, Fabio Fiordaliso, Luisa Diomede, Mario Salmona, Marco Gobbi

**Affiliations:** 1 Department of Molecular Biochemistry and Pharmacology, Istituto di Ricerche Farmacologiche Mario Negri IRCCS, Milan, Italy; 2 Department of Cardiovascular Medicine, Istituto di Ricerche Farmacologiche Mario Negri IRCCS, Milan, Italy

**Keywords:** Alzheimer's disease, amyloid-beta (Aβ), kinetics of fibril formation, tau, *Caenorhabditis elegans*, surface plasmon resonance, Aβ, amyloid-beta, AD, Alzheimer's disease, CB, Coomassie blue, PB, phosphate buffer, SPR, surface plasmon resonance, TEM, transmission electron microscopy, ThT, thioflavine T, WB, Western blot

## Abstract

The formation of neurofibrillary tangles and amyloid plaques accompanies the progression of Alzheimer's disease. Tangles are made of fibrillar aggregates formed by the microtubule-associated protein tau, whereas plaques comprise fibrillar forms of amyloid-beta (Aβ). Both form toxic oligomers during aggregation and are thought to interact synergistically to each promote the accumulation of the other. Recent *in vitro* studies have suggested that the monomeric nonphosphorylated full-length tau protein hinders the aggregation of Aβ_1–40_ peptide, but whether the same is true for the more aggregation-prone Aβ_1–42_ was not determined. We used *in vitro* and *in vivo* techniques to explore this question. We have monitored the aggregation kinetics of Aβ_1–42_ by thioflavine T fluorescence in the presence or the absence of different concentrations of nonphosphorylated tau. We observed that elongation of Aβ_1–42_ fibrils was inhibited by tau in a dose-dependent manner. Interestingly, the fibrils were structurally different in the presence of tau but did not incorporate tau. Surface plasmon resonance indicated that tau monomers bound to Aβ_1–42_ oligomers (but not monomers) and hindered their interaction with the anti-Aβ antibody 4G8, suggesting that tau binds to the hydrophobic central core of Aβ recognized by 4G8. Tau monomers also antagonized the toxic effects of Aβ oligomers in *Caenorhabditis elegans.* This suggests that nonphosphorylated tau might have a neuroprotective effect by binding Aβ_1–42_ oligomers formed during the aggregation and shielding their hydrophobic patches.

Alzheimer's disease (AD) affects 15% of individuals over the age of 65 years (1). It has a heavy sociohealth impact, partly related to increased life expectancy. Despite the scientific community's effort, no treatment is currently available. Most of the therapies tested so far focused mainly on amyloid-β (Aβ), small peptides of around 39 to 43 amino acids obtained by protease-mediated cleavage of the amyloid precursor protein (2). These peptides are found in amyloid deposits (plaques), one of the main pathological hallmarks of AD (3).

Especially abundant are two peptides: Aβ_1–40_ and Aβ_1–42_. The aggregation of these peptides, particularly by Aβ_1–42_, is thought to be the most toxic fragment leading to the neuronal damage in AD. However, most treatments focused on the “Amyloid hypothesis” have not been successful (4, 5); which is why other therapeutic targets too have been investigated (6, 7). Recent data suggest the importance of tau, the main component of the neurofibrillary tangles (8), that are another hallmark of AD.

Tau is a family of microtubules-stabilizing proteins made up of 352 to 441 residues, which are abundant in the central nervous system (7, 9). *In vivo* and *in vitro* data suggest that tau and Aβ interactions mutually influence the aggregation and toxicity of both molecules in AD (10, 11, 12). This could be because of not only fibrillar and oligomeric Aβ peptides inducing tau hyperphosphorylation, which could lead to loss of tau's microtubule-binding activity and neuron degeneration (12), but also the interaction of nonphosphorylated tau with Aβ_1–40_, which slowed the kinetics of fibril formation *in vitro* (12).

We investigated a possible direct interaction between tau monomers and Aβ_1–42_, the most toxic variant of the amyloid precursor protein cleavage products, its impact on the various stages of Aβ_1–42_ kinetics of fibril and oligomer formation, and on its *in vivo* toxicity. We used (i) thioflavine T (ThT) to investigate the kinetics of fibril formation in the presence of tau and employed protocols for analyzing the changes in the underlying molecular mechanisms of the aggregation process; (ii) surface plasmon resonance (SPR) to study the direct interaction between tau (2N4R) and Aβ_1–42_ monomers and oligomers and the changes in oligomer binding to anti-Aβ antibodies in the presence of tau; (iii) transmission electron microscopy (TEM), SDS-PAGE, and Western blot (WB) to further investigate the structure and composition of aggregation products; and (iv) a *Caenorhabditis elegans–*based toxicity assay, which has been used to detect toxic soluble assemblies of amyloidogenic proteins (13, 14).

## Results

### Tau inhibits Aβ_1–42_ fibrillogenesis

We used a ThT-based aggregation assay to determine the influence of tau on the kinetics and the underlying molecular mechanisms of Aβ_1–42_ fibril formation (15).

**Figure 1:**
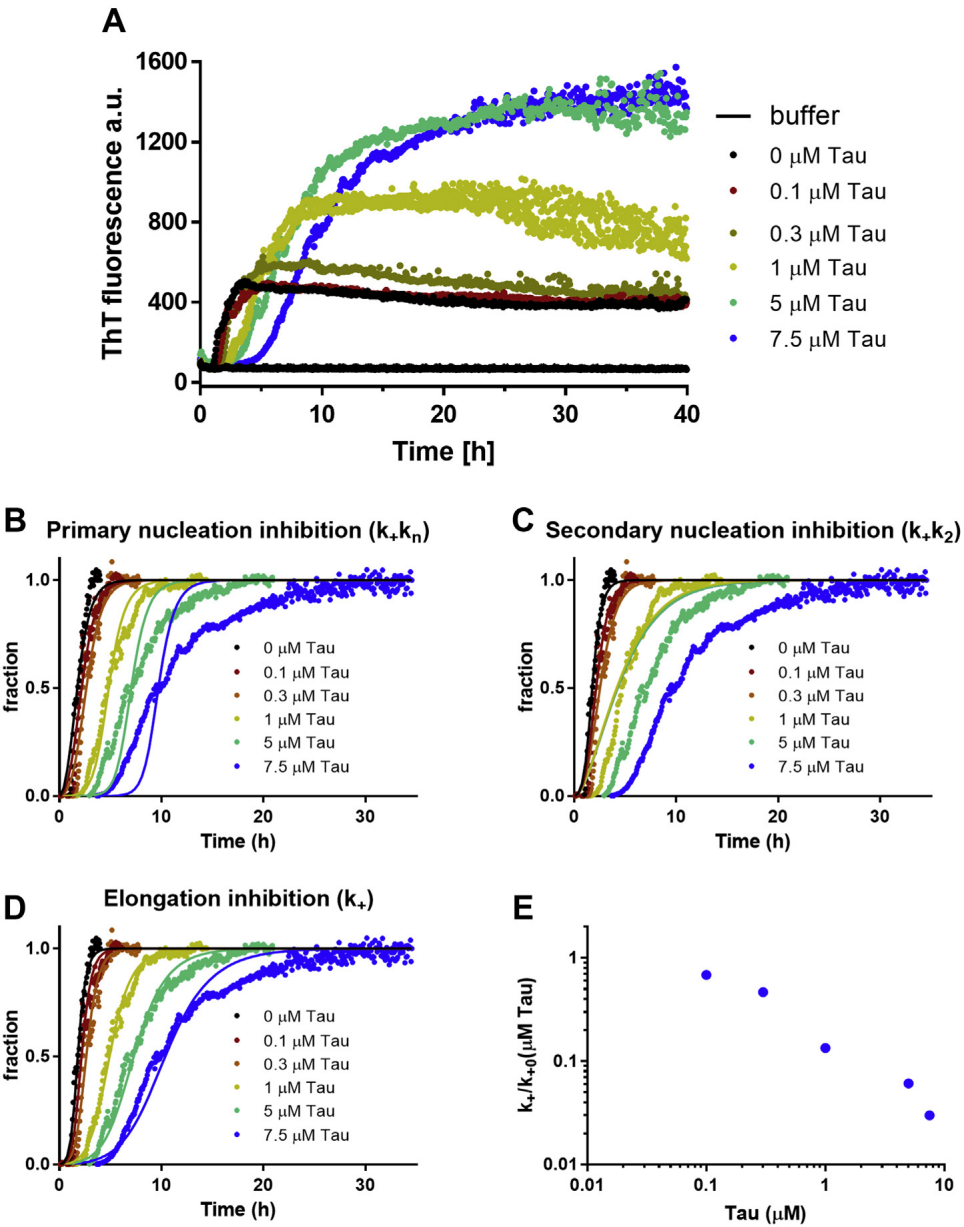
**Effect of monomeric tau on Aβ**_**1–42**_**fibril formation monitored by thioflavine T (ThT) fluorescence.***A*, time course of 5 μM Aβ_1–42_ fibril formation incubated, at 37 °C under quiescent conditions, with or without 0.1 to 7.5 μM tau in PBS obtained from two independent experiments run in triplicate, pH 7.4, and 20 μM ThT. *B*–*D*, normalized reaction curves showing the best global fits obtained with AmyloFit to the data varying the kinetic constants for (*B*) primary nucleation, (*C*) secondary nucleation, and (*D*) elongation. *E*, effective rate constants of elongation during Aβ_1–42_ aggregation, derived from (*D*). Aβ, amyloid-beta.

Five micromolars of freshly prepared Aβ_1–42_ were incubated in the absence and presence of five tau concentrations ranging from 0.1 to 7.5 μM in PBS at 37 °C, under quiescent conditions. The solutions contained 20 μM ThT, and the fluorescence intensity was measured every 5 min for up to 30 h. Nonphosphorylated tau delayed Aβ_1–42_ polymerization in a concentration-dependent manner (Fig. 1). The half-time of transition (50% of the Aβ monomer is converted into fibrils) rises from 2 h in the absence of tau to 10 h with 7.5 μM tau.

We then made a quantitative analysis of the effects of tau on the kinetics of Aβ_1–42_ fibril formation by fitting integrated rate laws to the aggregation traces (Fig. 1, *A*–*C*) (15, 16, 17). We perturbed previously determined rate constants of the dominating process (surface-induced secondary nucleation) (16) to identify the microscopic step (elongation [*k*_+_], primary nucleation [*k*_*n*_], and secondary nucleation [*k*_2_]) affected by the presence of tau. This showed that the elongation rate (Fig. 1*C*) was particularly perturbed since only a reduction of this rate can explain the experimental results. The elongation rate constant was lowered by approximately two orders of magnitude compared with that in the absence of tau (*k*_+0_) (Fig. 1*D*).

**Figure 2:**
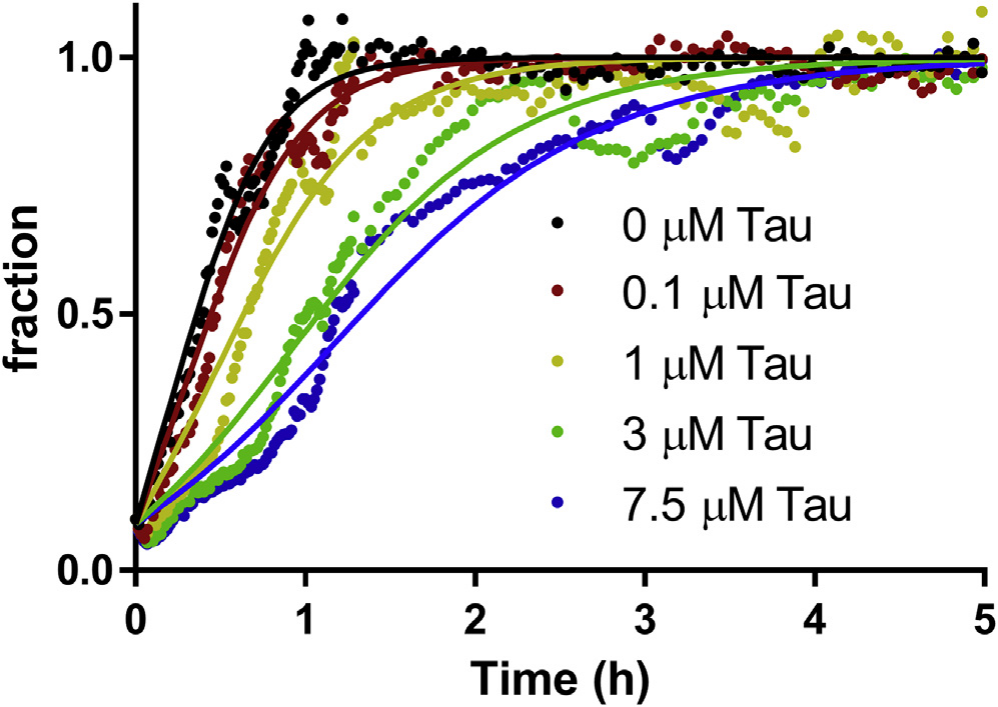
**Tau significantly inhibits elongation of Aβ**_**1–42**_**fibril formation.** Kinetic profiles of 5 μM Aβ_1–42_ aggregation with 10% preformed seeds were obtained in with or without (0.1–7.5 μM) in PBS, pH 7.4, and 20 μM thioflavine T (ThT), at 37 °C under quiescent conditions. *Solid lines* are fits of the reaction profiles when the elongation rate is allowed to vary. Aβ, amyloid-beta.

This was further confirmed in experiments under the same conditions as before but with 10% (mass equivalent) of preformed sonicated Aβ_1–42_ fibrils. In this condition, the nucleation steps were bypassed, and the dominant process for the consumption of monomers was the elongation. Tau significantly affected the aggregation kinetics (Fig. 2), and the experimental aggregation curves are described well by the mathematical model, when the elongation rate is lowered (Fig. 2).

### Tau catalyzes structural changes of Aβ_1–42_ fibril

**Figure 3:**
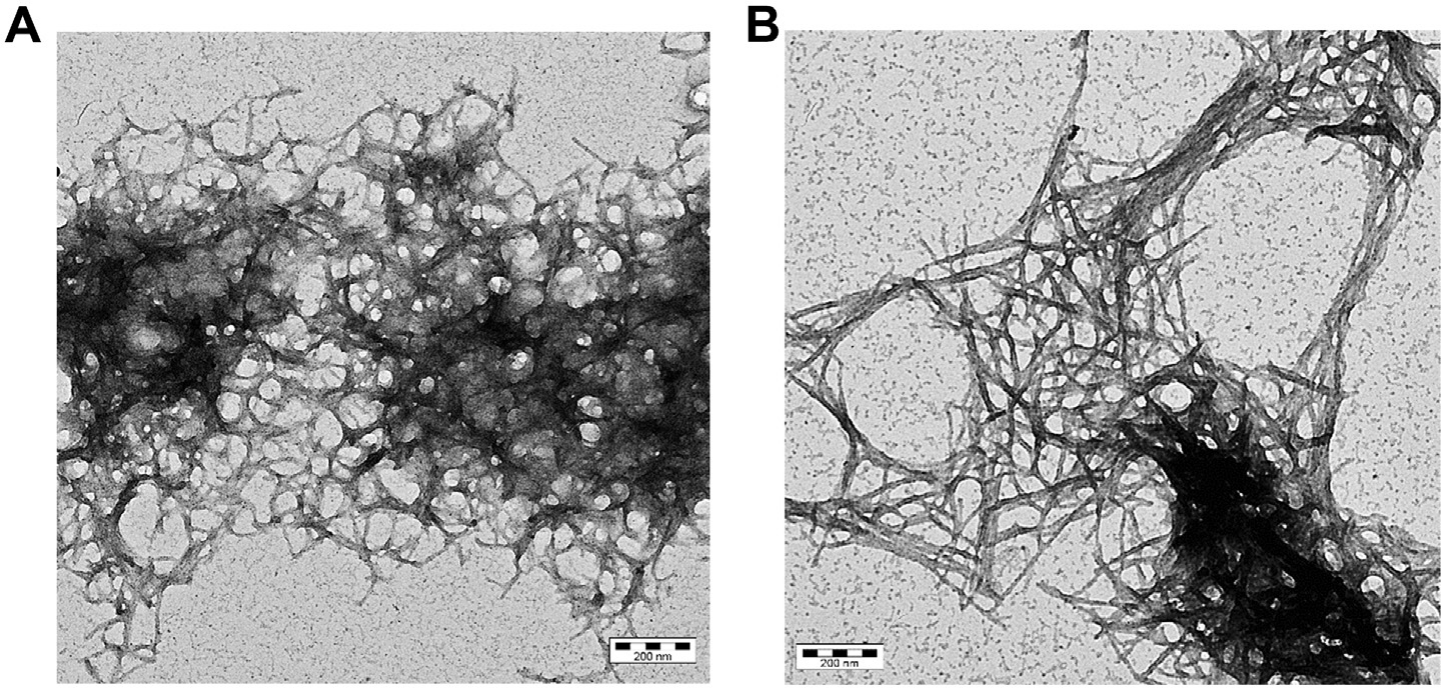
**Tau protein changes Aβ fibril structure.** Transmission electron microscopy images of (*A*) 5 μM Aβ_1–42_ alone and (*B*) with 5 μM tau at the end of the aggregation reaction (see Fig. 1) (bar length, 200 nm). Aβ, amyloid-beta.

The reaction curve of non-normalized ThT values from Figure 1*A* showed that the highest ThT value rose three times during the fibril formation of Aβ_1–42_ in the presence of 5 μM tau compared with Aβ_1–42_ alone (Fig. 1*A*). This might be due to changes in the fibril structure, which could increase the number of ThT-binding sites. Alternatively, more fibrillary material might form as a result of Aβ–tau heteroaggregation or the formation of tau fibrils. First, we used TEM to determine the morphology of the fibrils formed during the aggregation of 5 μM Aβ_1–42_ with and without 5 μM of tau for 48 h (Fig. 3, *A* and *B*). The aggregates formed in the presence of tau were longer, thicker, and straighter (Fig. 3*B*) than the fibrillary aggregates in the solution only containing Aβ_1–42_ (Fig. 3*A*).

**Figure 4:**
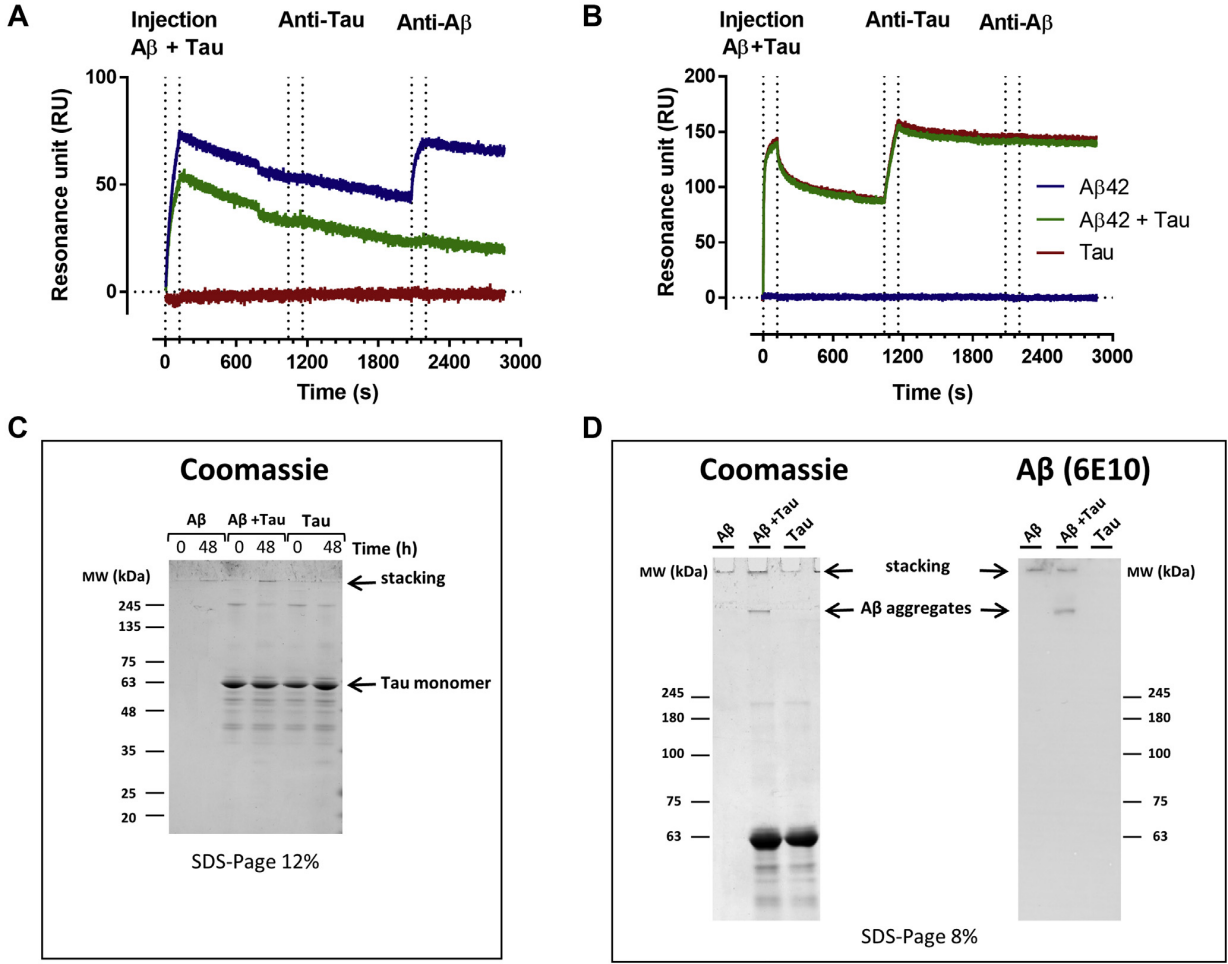
**Aβ**_**1–42**_**aggregates do not contain tau protein.***A* and *B*, surface plasmon resonance sandwich assay to analyze the composition of fibrillar aggregates of Aβ_1–42_ with and without tau and monomeric tau after 48 h. Solutions were flowed over immobilized (*A*) anti-Aβ 6E10 and (*B*) antitau Dako antibody, then followed by a second and third injection (sandwich) using antitau and anti-Aβ, respectively. *C*, solutions of 5 μM tau in 10 mM PBS, pH 7.4, 5 μM Aβ_1–42_, and 5 μM Aβ_1–42_ plus 5 μM tau in 10 mM PBS, pH 7.4, were analyzed before (0 h) and after (48 h) incubation at 37 °C in 12% SDS-PAGE and stained with Coomassie *blue*. *D*, the same solutions were analyzed 48 h after incubation at 37 °C in 8% SDS-PAGE and stained with Coomassie *blue* or immunoblotted with anti-Aβ 6E10 antibody. Aβ, amyloid-beta.

We used an SPR sandwich assay to clarify whether heterogeneous Aβ–tau aggregates were formed. The solutions used in the TEM experiment and tau alone, incubated for 48 h, were flowed first over anti-Aβ (Fig. 4*A*) or antitau (Fig. 4*B*) antibodies immobilized on the chip surface. The anti-Aβ antibody (6E10) captured Aβ_1–42_ species, either with or without tau, but no binding was detected flowing tau alone, as expected (Fig. 4*A*). Subsequent injection of the antitau secondary antibody gave no binding signal for any of the solutions suggesting that the Aβ_1–42_ species captured by immobilized 6E10 did not contain tau. A subsequent third injection of 6E10 gave a binding signal on the lanes where Aβ species were captured previously (*blue line*), confirming that they are Aβ oligomers. However, 6E10 did not detect the Aβ species captured by immobilized 6E10 after injection of the tau–Aβ mixture. Again, this might be due to the structural differences of Aβ_1–42_ aggregates, preventing their binding to 6E10, so that only the binding of Aβ monomers is detected under this condition.

Figure 4*A* shows that the antitau antibody (Dako) captured tau, either with or without Aβ, whereas no binding was detected with Aβ alone. The species in the tau–Aβ mixture captured by the antitau antibody do not appear to contain Aβ_1–42_, since 6E10 injected as secondary antibody did not cause any binding signal. The similarity of the sensorgrams and the absence of any ThT signal with tau alone suggest there is a similar amount of monomeric tau in the solution and that Aβ_1–42_ does not induce the formation of tau aggregates.

This was further supported by SDS-PAGE separation followed by Coomassie blue (CB) staining and/or Western blot (WB). The solution containing tau alone or Aβ with tau at time 0 and after 48 h had the same intense tau monomer band at 63 kDa in the CB-stained 12% gel (Fig. 4*C*). The separation and identification of Aβ_1–42_ aggregates after 48 h was possible with 8% SDS-PAGE gel and CB/WB (Fig. 4*D*). A notable amount of Aβ_1–42_ aggregates was situated in the stacking gel of Aβ_1–42_-containing solutions incubated with and without tau. When tau was present during the aggregation, there was an additional high–molecular-weight band recognized by the anti-Aβ 6E10 antibody (Fig. 4*D*), further supporting the formation of different Aβ_1–42_ aggregates in the presence of tau.

### Aβ_1–42_ high–molecular-weight oligomers but not monomers bind to tau monomers

SPR was then used to further study the direct interaction between different species of Aβ_1–42_ and tau protein.

**Figure 5:**
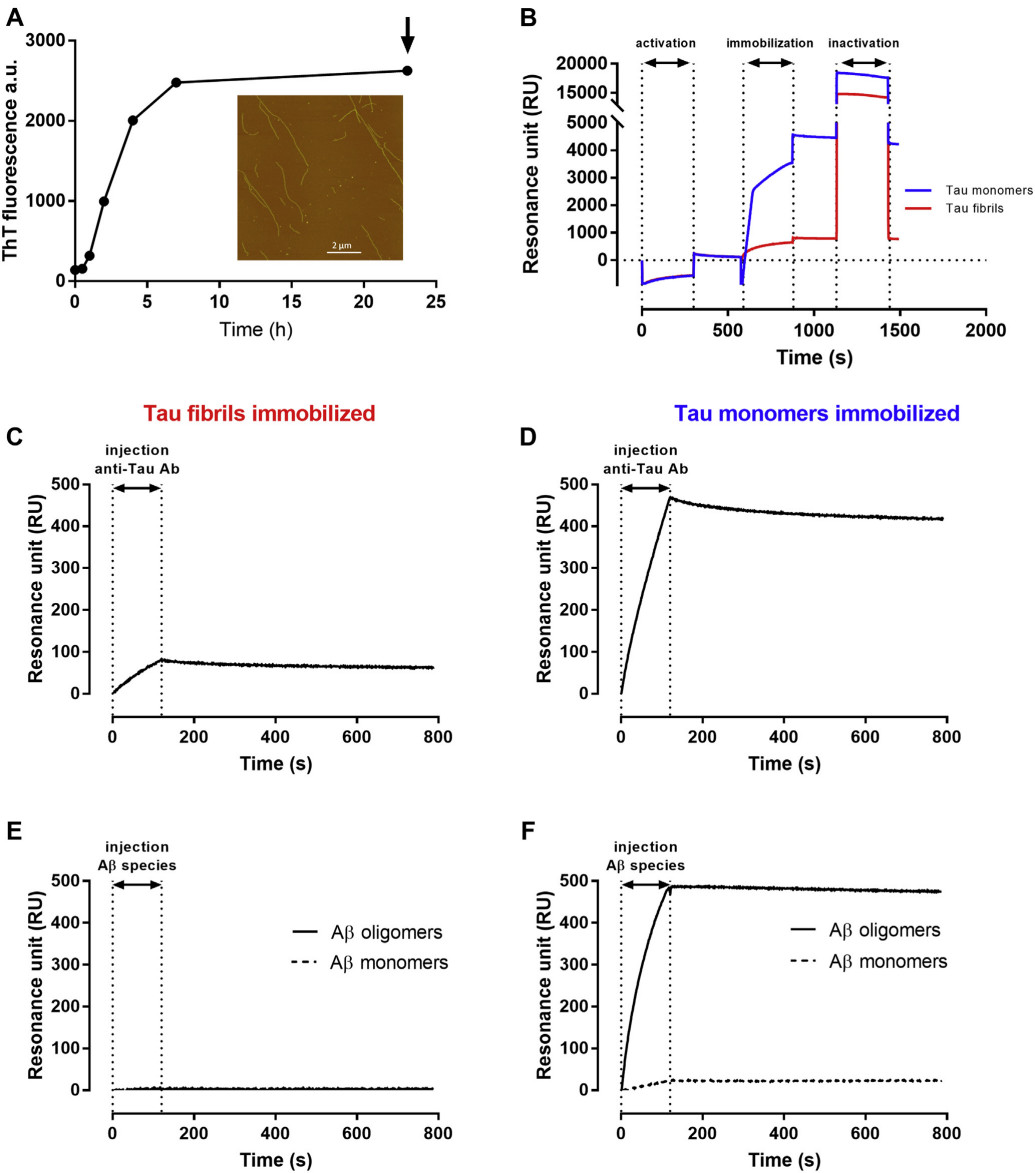
**Interaction between Aβ**_**1–42**_**monomers and oligomers with tau monomers and fibrils.***A*, time course of tau fibril formation was monitored by thioflavine T (ThT) fluorescence. The formation of fibrils was confirmed by atomic force microscopy (inset). *B*, immobilization of tau monomers (*blue line*) and tau fibrils (*red line*) on the surface plasmon resonance (SPR) chip. Graph shows the combined sensorgrams of the activation, immobilization, and inactivation steps. *C* and *D*, SPR sensorgrams obtained after injection of 100 nM antitau antibody A0024 over immobilized tau monomers and fibrils. *E* and *F*, SPR sensorgrams obtained after injection of Aβ_1–42_ monomers or oligomers over immobilized tau monomers and fibrils. For this, aliquots of freshly dissolved Aβ_1–42_ (100 μM) were diluted to 10 μM (monomers, *dashed line*) or incubated for 1 h at 37 °C in 10 mM PBS, pH 7.4, diluted to 10 μM (oligomers, *continuous line*), and injected for 120 s as indicated. Aβ, amyloid-beta.

Tau fibrils were formed by incubating 50 μM tau in 50 mM phosphate buffer (PB) at pH 7.4 in the presence of heparin and 1 mM dichlorodiphenyltrichloroethane at 37 °C. The kinetics of fibril formation was monitored by ThT fluorescence, which showed that the plateau was reached after 23 h (Fig. 5*A*). The formation of fibrils was confirmed by atomic force microscopy, which showed straight and several micrometer-long fibrils (Fig. 5*A*, inset). Tau monomers and fibrils were then immobilized in parallel surfaces of an SPR chip (Fig. 5*B*), at immobilization levels of 4200 and 770 resonance unit (RU), respectively. The following injection of the antitau antibody A0024 resulted in maximum binding signals of ∼480 and ∼80 RU on immobilized tau monomers and fibrils, respectively (Fig. 5, *C* and *D*), in agreement with the immobilization level (Fig. 5*B*).

We then analyzed the direct interaction of Aβ_1–42_ monomers and oligomers with tau monomers and fibrils. Previous studies showed that toxic high–molecular-weight aggregates are formed during the incubation of highly concentrated Aβ_1–42_ solutions (13, 18). These aggregates are short and worm like and detected by the OC antibody, specific for fibrillar oligomers (19). The enrichment of these on-pathway oligomers permits further study of their interactions with other proteins (16, 20) or inhibitors (13). For this, 100 μM of Aβ_1–42_ in PBS, pH 7.4, was incubated for 1 h at 37 °C. The oligomer-rich solution and an aliquot of the same solution taken at the start of the aggregation reaction was then diluted to 10 μM and injected over tau monomers and fibrils immobilized on the chip surface. No binding for either of the 2 Aβ_1–42_ solutions was observed for the tau fibrils (Fig. 5*E*). Oligomers, but not monomers of Aβ_1–42_, bound with high affinity to the tau monomers (Fig. 5*F*). The results shown in Figure 5*F* were replicated in three additional independent experiments (data not shown).

### Tau monomers inhibit the binding of synthetic Aβ_1–42_ high–molecular-weight oligomers to an Aβ-specific antibody

We then examined whether tau monomers binding to Aβ_1–42_ oligomers prevented the binding behavior of the latter. We exploited an SPR-based immunoassay, which can distinguish and quantify the specific binding of Aβ monomers and oligomers to immobilized 4G8 (13, 16).

**Figure 6:**
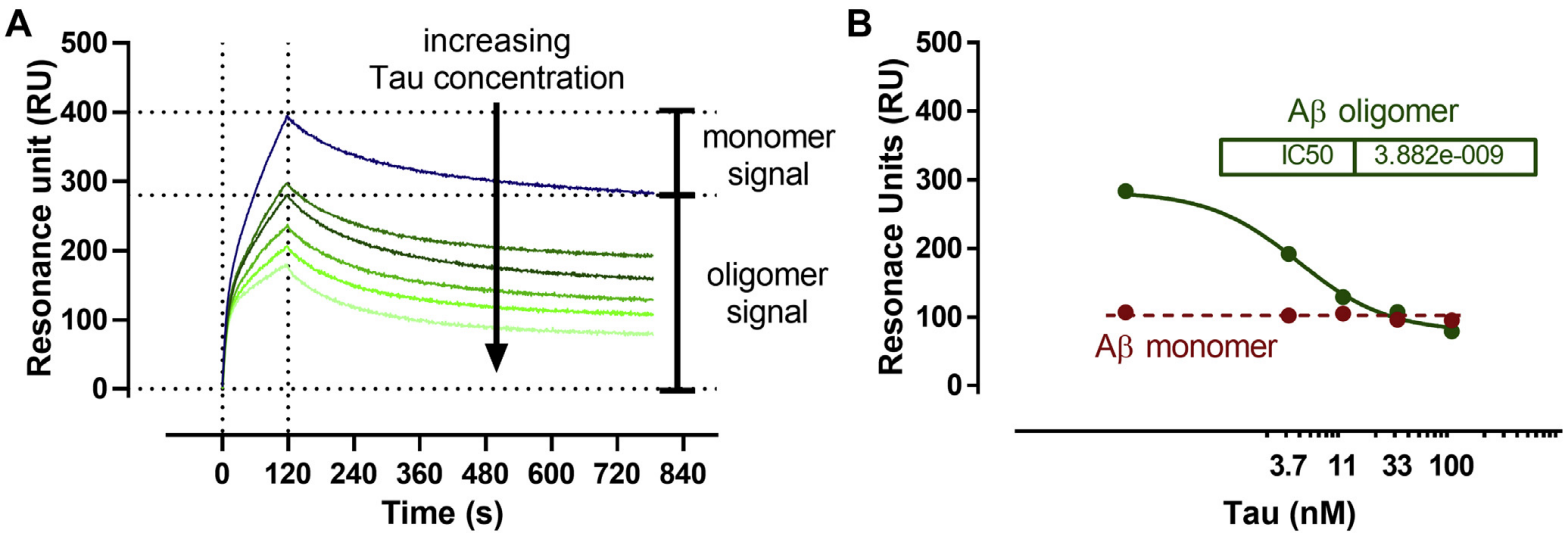
**Tau inhibits the binding of Aβ**_**1–42**_**oligomers to 4G8 antibody.** Preformed Aβ_1–42_ oligomers (100 μM) were diluted to 1 μM in 10 mM PBS, pH 7.4 containing 0, 3, 10, 30, and 100 nM tau, incubated for 10 min and injected into an surface plasmon resonance (SPR) apparatus for 120 s over immobilized anti-Aβ 4G8 antibody. *A*, tau concentration-dependent SPR signal. The sensorgram has two components; the fast-dissociating monomer, which is completely removed at the end of the dissociation (difference between SPR value at *t* = 120 s and *t* = 785 s) and the slow-dissociating oligomers (SPR value at *t* = 785 s). *B*, tau concentration–dependent SPR signals attributed to Aβ_1–42_ monomers (*red*) and Aβ_1–42_ oligomers (*green*). The IC_50_ is 3.9 nM. Aβ, amyloid-beta.

Aβ_1–42_ oligomer-enriched solutions were diluted to 1 μM into PBS and incubated with or without different tau concentrations (0.1–100 nM). After 10 min, the solutions were injected in parallel over 4G8 immobilized on the chip surface. The presence of tau reduced the SPR signal induced by Aβ_1–42_ oligomer, in a concentration-dependent manner (Fig. 6*A*). The deconvolution of the sensorgrams into monomer- and oligomer-specific signals (13) indicates that tau specifically affected oligomer-dependent binding, with an IC_50_ of 3.9 nM, with no change in the monomer-dependent signal (Fig. 6*B*).

### Tau reduces the toxicity of Aβ_1–42_ oligomers in *C. elegans*

**Figure 7:**
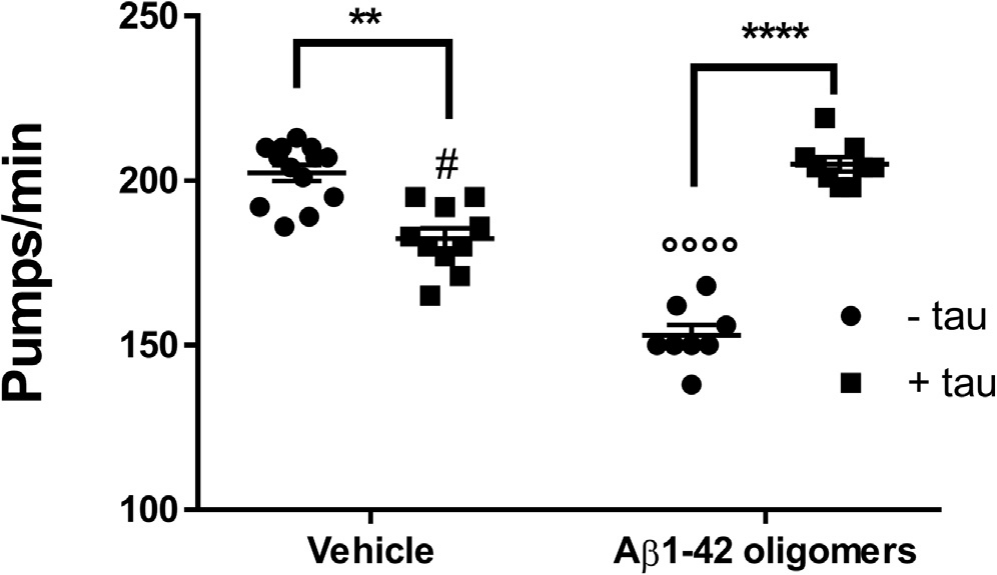
**Effect of tau on the toxicity of Aβ**_**1–42**_**oligomers in *Caenorhabditis elegans*.** Pharyngeal pumping (pumps/minute) of worms treated for 2 h with Aβ_1–42_ oligomers (10 μM), 100 nM monomeric tau, or 10 μM Aβ_1–42_ preformed oligomers in the presence of 100 nM tau. 10 mM PBS, pH 7.4, was used as negative control. ∗∗*p* < 0.01 and ∗∗∗∗*p* < 0.001, interaction Aβ_1–42_/tau = *p* < 0.0001, by two-way ANOVA and Bonferroni's post hoc test. ºººº*p* < 0.0001 *versus* vehicle - tau and #*p* < 0.5 *versus* Aβ_1–42_ oligomers - tau, by one-way ANOVA and Bonferroni's post hoc test. Aβ, amyloid-beta.

We then investigated whether the binding of tau to Aβ oligomer reduced the toxicity *in vivo*, using the invertebrate nematode *C. elegans.* This nematode is often used in toxicity studies since its pharynx is sensitive to sublethal doses of chemical stressors like toxic oligomers (13, 14, 16, 21, 22). We already reported that the rhythmic contraction and relaxation of the pharyngeal muscle in *C. elegans*, termed “pumping rate,” was significantly impaired on exposing the nematodes to an Aβ_1–42_ oligomer–enriched solution but not monomers or fibrils (13). This toxic effect of 10 μM of Aβ_1–42_ oligomers was completely prevented by 100 nM of tau (Fig. 7).

## Discussion

Preclinical studies showed that Aβ and tau influence each other. For instance, Aβ aggregates accelerate phosphorylation and aggregation of tau (23), and tau inhibits the toxicity of Aβ aggregates (24), an effect possibly mediated by direct interaction between the two proteins (11, 12). The two monomeric forms of Aβ and nonphosphorylated tau may interact with each other in the extracellular space since both proteins are excreted by neurons (25, 26). A more recent fuller study (12) directly demonstrated that different forms of Aβ_1–40_ aggregates, but not monomers, bind to tau monomers with affinity constants ranging from 1 to 10 μM, respectively, for oligomers and fibrils, leading to the inhibition of kinetics of fibril formation.

We extended this work by using the more aggregation-prone and clinically more relevant fragment Aβ_1–42_. We found that tau slowed the amyloid fibril formation of Aβ_1–42_, as previously observed with Aβ_1–40_ (12). By analyzing the ThT measurements with a mathematical scheme that can distinguish the microscopic mechanisms of Aβ aggregation (17, 27), we found that the inhibitory effect of tau resulted from its ability to significantly slow elongation.

Although tau slows the kinetics of both Aβ_1–42_ and Aβ_1–40_ (12) fibrillogenesis, there was a clear difference between the 2 Aβ peptides with regard to the highest ThT signal, which was lowered for Aβ_1–40_ (12) and raised for Aβ_1–42_ (this study). This is possibly explained by the fact that tau favors the formation of amorphous Aβ_1–40_ aggregates (12), whereas for Aβ_1–42_, it induces the formation of more extended and straighter Aβ_1–42_ fibrils. Interestingly, in addition to the Aβ_1–42_ aggregates retained in the stacking gel of the SDS-PAGE, tau induced a second Aβ_1–42_ high–molecular-weight aggregate population. This might be due to differences in mobility in SDS-PAGE governed by the different aggregate morphology.

This interaction may occur to the end of the fibrillar aggregates of Aβ_1–42_ (28, 29), since the putative binding site in the C terminus of Aβ_1–42_ is buried in the fibril core (29, 30). Moreover, the observation that Aβ_1–42_ fibrillar aggregates bind to tau monomers and not to tau fibrils may suggest that the binding involves the hydrophobic hexapeptide motif paired helical filament-6 present in the R3 domain of tau (tau 306–311), which is incorporated into the fibril core and not accessible for binding (31).

This could be also relevant for phosphorylated tau, since no phosphorylation site is present in paired helical filament-6 (32, 33). Nevertheless, this interaction is not strong enough to form heterogeneous aggregates, since we did not find any evidence of incorporation of tau protein into the Aβ_1–42_ fibrils, as for other proteins (34). This was investigated in SPR sandwich assay and by separation of the different components in solution by SDS-PAGE. The SPR studies indicated that an antitau antibody does not recognize the Aβ_1–42_ aggregates obtained in the presence of tau. Moreover, we saw now wildtype tau protein aggregation induced by Aβ_1–42_, even though tau P301L fibril formation can be caused by Aβ (35). This could be due to the lower aggregation propensity of the tau wildtype protein compared with tau mutation P301L (36). This is supported by the unchanged monomer concentration of tau in SDS-PAGE and the similar SPR sensorgrams after exposure to solutions containing tau monomer preincubated or not with Aβ_1–42._

Specific inhibition of the microscopic pathways during the aggregation of Aβ_1–42_ might lead to changes in the formation of toxic oligomers (37). That is why we investigated the effects of tau on the binding properties and toxicity of Aβ_1–42_ oligomers. Fibrillar Aβ_1–42_ oligomers, but not monomers, bound to tau monomers. The sensorgrams enabled us to estimate affinity constants (*K*_*D*_) lower than 1 nM, assuming oligomer concentrations of 4 to 20 nM and an aggregate size of 400 kDa (13). This *K*_*D*_ is possibly overestimated since multivalent interactions (avidity) of the oligomers with the tau monomer might alter the apparent dissociation rate. In addition, the conformation of the tau protein might change because of covalent immobilization of the tau protein on the chip surface. This was circumvented by an SPR-based competition assay. Preincubation in solution, between Aβ_1–42_ and tau, prevented the Aβ_1–42_ oligomers (but not monomers) binding to immobilized 4G8 (13). The 4G8 antibody detects not only Aβ monomers but also fibrillar oligomers (13, 19, 38). In fact, the binding of Aβ_1–42_ monomers and oligomers to immobilized 4G8 can be distinguished, as previously described (13), because fibrillar oligomers bind with high affinity in a pseudoirreversible manner, whereas the monomers dissociate entirely in less than 10 min (13). Thus, the final signal is indicative of the number of oligomers bound to 4G8. The results indicated that tau prevented the binding of these oligomers to 4G8 with an IC_50_ of 3.8 nM, confirming the high-affinity binding of Aβ_1–42_ oligomers to the tau monomer indicated by direct SPR analysis. Binding of tau to the C terminus of fibrillar aggregates of Aβ_1–42_ *via* the hexapeptide motif may hinder the binding of 4G8 antibody to residues 18 to 24 of Aβ_1–42_. The affinity of tau for Aβ_1–42_ oligomers appears to be about three orders of magnitude greater than previously found with Aβ_1–40_ (12). The apparent discrepancy between the estimated binding affinity (<10 nM) and the higher concentration (>100 nM) needed to influence the kinetics of fibril formation might be due to a higher *K*_*D*_ value of early fibrillary oligomers. Also, in the case of Aβ_1–40_, the *K*_*D*_ changed during the kinetics of fibril formation. Species present at the beginning of the aggregation had a higher *K*_*D*_ value than the population formed later on (12).

Previous studies strongly suggest that the Aβ_1–42_ oligomers detected by 4G8 in the SPR assay are actually the toxic species (13). This was indicated by different approaches but in particular looking at the toxic effects on the physiological pharyngeal contractions in *C. elegans*, an *in vivo* assay (13, 16, 20, 39). We found that tau antagonized the toxic effects of Aβ_1–42_ oligomers on the worms' pharyngeal pumping rate. We suggest that this was due to the binding of tau to—and shielding of—the hydrophobic patches exposed on fibrillar Aβ_1–42_ oligomers (16, 40).

Even though most of the experimental evidence points to a synergistic effect of Aβ and tau in AD, our results suggest that nonphosphorylated tau might play a protective role, by slowing the aggregation of Aβ. The active concentrations used in these experiments are close to the overall brain concentration of tau (41, 42). Although phosphorylated tau is considered an important marker in the diagnosis of AD, the concentration of nonphosphorylated tau is nearly 10 times higher in AD brains (43, 44), enough to interfere with plaque formation *in vivo*.

Nevertheless, since the current study focuses on nonphosphorylated tau, we cannot exclude the possibility that phosphorylated tau could have a similar protective function.

In summary, our data suggest the potential role of nonphosphorylated tau monomers during Aβ_1–42_ fibril formation. The molecular properties underlying this interaction may lead to the development of new synthetic tau derivatives with potential therapeutic applications in AD.

## Experimental procedures

### Aβ_1–42_ synthesis and preparation

Aβ_1–42_ was synthesized in-house in its depsi form, as described previously (45). Depsi-Aβ_1–42_ has a reduced tendency to aggregate in acidic solutions (pH <3), so seed-free stock solutions of monomeric Aβ_1–42_ can be prepared. The concentration was determined by UV spectroscopy (ε_214 nm_ = 76,848 M^−1^ cm^−1^). The native form of Aβ_1–42_ was obtained by changing the pH of the solution to 7.4.

### Preparation of recombinant tau protein

Human wildtype tau protein was expressed and purified according to previously described protocols (46, 47, 48). First, the complementary DNA of the 441-amino acid tau isoform (2N4R) was cloned into the pRK172 bacterial expression vector and expressed in BL21(DE3) *Escherichia coli* bacteria (Novagen; Merck KGaA) with isopropyl-*β*-d-thiogalactopyranoside (1 mM for 2.5 h). The bacteria were then pelleted and dissolved in lysis buffer (20 mM piperazine-*N*,*N*′-bis(2-ethanesulfonic acid) at pH 6.8, 1 mM ethylene glycol-bis(2-aminoethylether)-*N*,*N*,*N*′*N*′-tetraacetic acid, 1 mM dithiothreitol, protease inhibitors cocktail) sonicated and centrifuged. The lysis was repeated two times. After lysis of the bacteria and the removal from the supernatant of nucleotides and heat-denatured proteins, the tau protein was purified by using cation column exchange. Fractions containing the highest concentration of protein were determined by SDS–PAGE, pooled and verified by WB using the mouse antitau (4-repeat isoform RD4) monoclonal antibody (1:2000 o/n, clone 1E1/A6, 05-804; Merck), followed by antimouse IgG peroxidase conjugate antibody (1:5000, 2 h, A9044; Sigma). The concentration was determined by densitometric analysis (Image Lab 6.0; Bio-Rad) of R-250 CB-stained SDS polyacrylamide gels in comparison to commercial tau protein as standard (ab84700; Abcam).

### Kinetics of fibril formation

The kinetics of fibril formation was measured in an *in situ* ThT fluorescence assay (16, 49). Briefly, 5 μM Aβ_1–42_ fibril formation was monitored with or without different concentrations of tau (0.1–100 nM) in 10 mM PBS, pH 7.4, and 20 μM ThT under quiescent conditions at 37 °C in microplate wells (Microplate Corning 3651, 96 wells, low binding; Corning Incorporated Life Sciences). ThT fluorescence was measured every 5 min with an F500 Infinity plate reader (Tecan Italia Srl) using 440 nm for excitation and 495 nm as emission wavelength.

The global analysis of the normalized ThT data (using the maximal ThT value of the reaction curves for each single condition) was used to determine which microscopic assembly processes were affected by the presence of tau. This analysis is based on the integrated rate laws describing the evolution of total fibril mass *M*(*t*) in the presence of primary and possibly secondary nucleation events (17, 27, 49, 50, 51):

(1) $$\frac{M\left(t\right)}{M\left(\infty \right)}=1-\alpha {\left(\frac{B_{+}+C_{+}}{B_{+}+C_{+}e^{\kappa t}}\frac{B_{-}+C_{+}e^{\kappa t}}{B_{-}+C_{+}}\right)}^{\frac{\kappa_{\infty }^{2}}{\kappa {\tilde{\kappa }}_{\infty }}}e^{-\kappa_{\infty }t}$$

The parameters $B_{\pm }$, $C_{\pm }$, κ, $\kappa_{\infty }$, and ${\tilde{\kappa }}_{\infty }$ are functions of combinations of the microscopic rate constants *k*_+_*k*_2_ and k_*n*_k_2_, where k_*n*_, k_+_, and k_2_ are the primary nucleation rate, elongation rate, and secondary nucleation rate constants, respectively, and the reaction orders *n*_c_ and *n*_2_, describing the dependency of the primary and secondary pathways on the initial monomer concentration (16). One of these microscopic processes mostly dominates the kinetics, and for our synthetic Aβ_1–42_, it is surface-induced secondary nucleation (16). We used the previously determined rate constants to constrain the global fitting, leaving free the rates free to change only when tau was present. The reaction curves were fitted using the online platform AmyloFit (https://www.amylofit.ch.cam.ac.uk/) (52). The influence of tau on the kinetics of fibril Aβ_1–42_ formation was repeated by adding 10% (mass equivalent) of preformed sonicated Aβ_1–42_ fibrils. These seeds were made by incubating 5 μM Aβ_1–42_ in 10 mM in PBS for 5 h. Fibrils were broken by probe sonication on ice using 6 × 10 s pulses.

### Preparation of Aβ_1–42_ oligomer-enriched solution

The solution was prepared by diluting depsi-Aβ_1–42_ stock solution in 10 mM PBS (pH 7.4) to 100 μM. Oligomer-enriched solutions were obtained by incubating this solution for 1 h at 37 °C.

### Preparation and characterization of tau fibrils

Tau fibrils were obtained by incubating 50 μM of the tau protein in 50 mM PB at pH 7.4 in the presence of 1 mM dichlorodiphenyltrichloroethane and heparin at a molar ratio of 1:4 to tau for 23 h at 37 °C (53, 54). The kinetics of tau fibril formation was monitored by measuring the ThT fluorescence at different time points. For these, aliquots of the sample were diluted into 50 mM PB at pH 7.4 containing 20 μM ThT and transferred to a microplate. The F500 Infinity plate reader from Tecan was used to determine the ThT emission at 495 nm after the excitation at 440 nm. The fibril formation was also confirmed by atomic force microscopy. The sample was diluted in 50 mM PB at pH 7.4 to a concentration of 10 μM and incubated for 2 min on the surface of a freshly cleaved mica disk. The disk was rinsed with water, and the surface tried under a gentle nitrogen stream. The sample was mounted on a Nanoscope V instrument (Veeco, Digital Instruments). The surface was scanned using tapping mode and a standard phosphorus-doped silicium probe (Bruker).

### Semidenaturing gel electrophoresis and WB

To characterize the protein solution, 5 to 20 μl of each were diluted in loading buffer (25 mM Tris, 200 mM glycine, 0.2% SDS, 5% glycerol, and 0.025% bromophenol blue), incubated for 7 min, then separated in 2.5% stacking and 8% or 12% resolving polyacrylamide gels. At the end of the electrophoresis, the gels were electroblotted onto a polyvinylidene fluoride membrane or stained with R-250 CB, followed by a blocking step and incubation of the membrane with mouse anti-Aβ monoclonal antibody 6E10 (1:2000 o/n, SIG-39320-200; Covance). An antimouse IgG peroxidase conjugate (1:5000, 2 h, A9044; Sigma) was used to detect bound 6E10 by chemiluminescence (ChemiDoc; Bio-Rad).

### SPR

We used the ProteOn XPR36 (Bio-Rad Laboratories) equipped with a GLC sensor chip for the SPR experiments. We employed amine-coupling chemistry to bind the ligands to the chip's surface, as described previously (13, 16). The immobilization level of the various proteins during these different experiments was 4400 RU for tau monomers, 770 RU for tau fibrils, 5800 RU mouse anti-Aβ monoclonal antibody 6E10, 5900 RU for rabbit antitau polyclonal antibody A0024 (Dako), and 4700 RU for mouse anti-Aβ monoclonal antibody 4G8 (1 RU corresponds to 1 pg protein/mm^2^). In each experiment, a control surface was used: this was an “empty” surface (activation/deactivation cycle) for immobilized tau proteins or an IgG mix for antibodies.

After the immobilization step, the microfluidic system was rotated, and the analytes were injected for 120 to 180 s at a flow rate of 30 μl/min. Generally, Aβ_1–42_ solutions were diluted to 1 μM in running buffer and injected over the different ligands. The running buffer was 10 mM PBS containing 150 mM NaCl and 0.005% Tween-20. The inhibiting effect of tau on Aβ_1–42_ binding to the 4G8 antibody was tested by incubating an oligomer-enriched solution of Aβ_1–42_ containing different concentrations of recombinant tau (1.2, 3.7, 11, 33, and 100 nM) for 10 min and injecting the samples in parallel on the immobilized antibody. The SPR signals on the chip surfaces were normalized to a baseline value of zero and corrected by subtracting the nonspecific response on the reference surface.

### TEM

Aβ_1–42_ aggregates incubated with or without 5 μM tau were placed on a 100 mesh formvar/carbon-coated copper grid (EMS) and left to dry at room temperature for 30 min. Grids were washed three times with water, counterstained with 2% uranyl acetate, and observed with an Energy Filter Transmission Electron Microscope (ZEISS LIBRA 120) equipped with a yttrium aluminum garnet scintillator slow-scan charge coupled device camera.

### Aβ_1–42_ oligomer toxicity

The procedure described by Stravalaci *et al*. (13) was used. Briefly, N2 nematodes (Caenorhabditis Genetic Center) were propagated at 20 °C on solid nematode growth medium seeded with *E. coli* OP50 (Caenorhabditis Genetic Center) for food. At the L3–L4 larval stage, worms were collected with M9 buffer, centrifuged, and washed twice with 5 mM PBS, pH 7.4, to eliminate bacteria. Worms (100/100 μl) were exposed to oligomer-containing solutions previously incubated with or without 100 nM tau for 2 h at 37 °C. No *E. coli* was present during the preincubation to avoid any potential interference with the peptides. Control worms were incubated with 5 mM PBS, pH 7.4 (vehicle), or tau alone. After 2 h of orbital shaking, the worms were transferred onto nematode growth medium plates seeded with OP50 *E. coli*. Pharyngeal pumping was scored 2 h later by counting the number of times the terminal bulb of the pharynx contracted in a 1-min interval (pumps/minute).

## Data availability

All data generated or analyzed during this study are included in this published article or available from the corresponding author upon request.

## Conflict of interest

The authors declare that they have no conflicts of interest with the contents of this article.
